# Prevalence and morphometric assessment of the middle mesial canal in mandibular first molars in a turkish population: A CBCT study

**DOI:** 10.1007/s10266-026-01325-3

**Published:** 2026-02-26

**Authors:** Elif Solakoğlu, Özge Kurt

**Affiliations:** 1 Department of Endodontics, Faculty of Dentistry, Bingöl University, Selahaddin-I Eyyübi neighborhood. Ayhan Çiftçi St. No: 1502, Bingöl, Turkey; 2 Department of Endodontics, Faculty of Dentistry, Aksaray University, Bahçesaray Neighborhood, Necmettin Erbakan Boulevard, Aksaray University Campus Road, 68100 Aksaray, Turkey

**Keywords:** Mandibular first molar, Middle mesial canal, Prevalence, Morphometric analysis, Cone-beam computed tomography

## Abstract

This study aimed to evaluate the configurations, prevalence, and morphometric relationships of the middle mesial canal (MMC) with surrounding structures in mandibular first molars (MFMs) of a Turkish population using cone-beam computed tomography (CBCT). CBCT images of 2,999 retrospectively selected MFMs were examined. The presence and configuration of the MMC and its relationship with the mesiobuccal (MB) and mesiolingual (ML) canals were assessed. In addition, the distances between the MB–ML, MB–MMC, and ML–MMC canal orifices and the vertical distances from the MB, ML, and MMC orifices to the pulpal floor (PF) were measured. Buccolingual (BL) and mesiodistal (MD) dimensions and their ratios were also analyzed in relation to the presence of the MMC. An MMC was detected in 24.5% of the examined teeth. Among these, 77.8% exhibited a configuration merging with the MB canal. The presence of an MMC was significantly associated with a smaller BL width and a larger MD width (*p* < 0.001). The MMC orifice was positioned deeper than the MB and ML orifices. In the Turkish population, MMCs were observed in 24.5% of MFMs, most commonly showing a merging configuration with the MB canal. The presence of an MMC can be predicted by specific morphometric parameters, and access can be facilitated by troughing between the MB and ML canals. These findings highlight the importance of anatomical awareness of the MMC to enhance clinical success.

## Introductıon

One of the most critical causes of endodontic treatment failure is the inability to completely eliminate microorganisms and infected tissues from the root canal system [1, 2]. In this inadequacy, undetected additional canals—which frequently arise due to the complex root canal morphology and anatomical variations—are reported to play a significant role [3, 4]. Jang et al*.* reported that undetected accessory canals were present in 21.7% of all cases and contributed to short-term treatment failure in up to 36.9% of cases [5].

Among teeth receiving endodontic therapy, the mandibular first molars (MFMs) are the most frequently treated [6, 7]. The mesial roots of MFMs typically contain two main root canals. In 1974, Barker and Vertucci first described a third canal, the middle mesial canal (MMC), positioned between the mesiobuccal (MB) and mesiolingual (ML) canals [1, 8]. Subsequent studies employing advanced imaging techniques have reported that the prevalence of MMCs in mandibular first and/or second molars varies widely across populations, ranging from 0.26% to 53.8% [9, 10]. This wide range and the morphological variability often lead to the canal being overlooked. Indeed, a CBCT-based study on endodontically treated teeth revealed that the MMC was not clinically detected in 97.53% of teeth in which it was present [11], emphasizing the importance of recognizing and addressing this canal to ensure endodontic success.

Several studies have demonstrated that morphometric analyses of the intracanalar distance between MB and ML canal orifices, as well as buccolingual (BL) and mesiodistal (MD) dimensions and their ratios, can provide clinically meaningful information [12–14]. Such measurements have been proposed as predictive indicators for the presence of an MMC and may assist in the clinical localization of its orifice [14]. In this context, Pomeranz classified MMCs within the mesial root of MFMs into three morphological configurations based on periapical radiographs: independent, confluent, and fin types. The independent type begins at a separate orifice and terminates at its own apical foramen; the confluent type merges with either the MB or ML canal at some point along the root; and the fin type involves a transversal connection between the MMC and one of the main mesial canals that allows instrument passage [15].

With the increasing use of cone-beam computed tomography (CBCT), Barros-Costa et al*.* refined this classification by further specifying MMCs merging with the MB canal as MB-confluent and those merging with the ML canal as ML-confluent, while excluding the fin type to distinguish MMCs from isthmus formations [13, 14].

Intraoral and panoramic radiographs, which have long been used in endodontics, are limited in complex cases due to geometric distortion and anatomical superimposition [16]. Although CBCT enables the three-dimensional examination of dentofacial structures [3], the current joint position statement by the AAE and AAOMR emphasizes that conventional radiography remains the primary diagnostic method. Accordingly, CBCT should not be used as a routine screening tool, but rather preferred only when conventional methods are insufficient or in cases involving anatomical complexity [17].

The clinical importance of the MMC lies in its contribution to the success of root canal therapy by facilitating thorough cleaning and shaping up to the obturation phase. While many studies have investigated MMC prevalence, Bascón-Mallado et al*.* not only assessed its frequency but also quantitatively measured the distance between the MB and ML canal orifices in teeth with an MMC, offering valuable guidance for localizing its position in clinical practice [12]. Similarly, Wang et al*.* reported dimensional parameters that may aid in predicting the location of the MMC [14]. Despite numerous CBCT-based investigations of MMC prevalence, detailed morphologic characteristics and spatial relationships between the MMC and adjacent mesial canals remain limited. Our study differs from previous research through its large sample size, population characteristics, the use of a contemporary MMC classification system, and the inclusion of multiple morphometric measurements, providing comprehensive and detailed data.

The present study aims to evaluate the prevalence and morphometric characteristics of the MMC in mandibular first molars in a Turkish population, with respect to sex, using CBCT. Specifically, it seeks to quantify the horizontal distances between the MMC and the MB and ML canal orifices, as well as the vertical relationship of these canals to the pulpal floor. We hypothesize that the presence of an MMC can be predicted using specific morphometric parameters. Such quantitative data may enable clinicians to approach the cleaning and shaping phases of treatment more strategically, ultimately enhancing treatment success.

## Materıals and methods

This study and its protocol were approved by the Scientific Research and Publication Ethics Committee of Bingöl University, Faculty of Health Sciences (Ethical Approval No: 2024/E.183017 Decision no:26), and all procedures were conducted in accordance with the principles of the Declaration of Helsinki.

### Study design and sample selection

Between August 2020 and August 2024, cone-beam computed tomography (CBCT) images of Turkish patients aged 18 to 55 years were retrospectively retrieved from the database of a private dental clinic. Written informed consent was obtained from all patients to allow the use of their CBCT data for research purposes. All CBCT scans included in this study had originally been acquired for clinical diagnostic and treatment planning purposes such as facial trauma, maxillary sinus evaluation, oral surgery, orthodontic or endodontic assessment, or implant planning—not specifically for research.

### Imaging parameters

CBCT images were obtained using a NewTom 5G CBCT unit (QR srl, Verona, Italy) operating with an 8 × 8 cm field of view (FOV), 200 µm (0.20 mm) voxel size, 60–90 kVp, and 6–15 mA settings to balance diagnostic accuracy and radiation exposure [18, 19]. The exposure time, automatically adjusted to the scanning area, was approximately 7 s for a full-arch scan. CBCT data were exported in DICOM format and analyzed using NNT Viewer software (QR srl, Verona, Italy) on a monitor with a resolution of 1920 × 1080 pixels.

### Sample size calculation

Previous studies identified small effect sizes for various morphometric parameters [14, 20]. Using G*Power 3.1, a minimum sample size of 1300 cases was calculated to achieve 95% power (*α* = 0.05) for a Chi-square test (goodness-of-fit, Cohen’s *w* = 0.1, *df* = 1) [20]. Consequently, CBCT images of 2000 patients were included, yielding 3,174 mandibular first molars (MFMs). After applying the inclusion and exclusion criteria, 2999 MFMs were selected for analysis.

### Inclusion criteria

1. Diagnostic quality CBCT images acquired with an 8 × 8 cm field of view (FOV) and 200 µm (0.20 mm) voxel size, allowing for reliable assessment of root canal morphology
2. Fully developed roots.

### Exclusion criteria

1. Teeth previously treated endodontically;
2. Teeth restored with crowns or post–core restorations;
3. Teeth with incomplete root development (immature apex);
4. Developmental anomalies (e.g., dens invaginatus, fusion, gemination);
5. Teeth with deformities, calcified canals, root resorption, or root fractures;
6. CBCT images were excluded if the presence, absence, or course of a round or oval radiolucent middle mesial canal could not be reliably assessed during a systematic inspection of axial, sagittal, and coronal sections from the pulp chamber floor to the apex, due to limitations in image quality (e.g., beam hardening, noise, motion, or other image artifacts) [14, 21].

All CBCT measurements were performed using Multi-Planar Reconstruction (MPR) images with standardized device and software settings. Anatomical reference points were identified manually in the plane most appropriate for each measurement parameter, and their positions were simultaneously verified in the orthogonal planes (axial, sagittal, and coronal) using the software’s synchronization feature. The distances between these manually determined points were calculated using the software's digital measurement tool. Although the software provided a digital readout precision of three decimal places, data were rounded to two decimal places for statistical analysis.

### Observer calibration and reliability assessment

Prior to image evaluation, a calibration session was conducted. To assess inter- and intra-observer reliability in the analysis of MMC prevalence, morphometric classification, and measurements, 30 CBCT scans were independently evaluated by two examiners blinded to each other’s assessments and to patient demographic data. Inter-observer agreement was calculated using Cohen’s kappa coefficient (*κ* = 0.768), while intra-observer agreement, determined by re-evaluation after two weeks, was *κ* = 0.754. Both values fell within the 0.61–0.80 range, indicating good qualitative reliability.

Quantitative reliability of the morphometric measurements was assessed using the Intraclass Correlation Coefficient (ICC) based on a two-way random-effects model (ICC [1, 2]) with absolute agreement. The intra-observer ICC was 0.72 (95% CI: 0.61–0.81), and the inter-observer ICC was 0.68 (95% CI: 0.56–0.78), reflecting moderate consistency and acceptable methodological reliability. All assessments were performed by two experienced endodontists; disagreements were resolved through a structured consensus process.

### Assessment of Middle Mesial Canal (MMC) prevalence

To minimize potential misinterpretation due to the complexity of the root canal system, the presence of MMCs in MFMs was evaluated by two investigators. Using the toolbar function, each tooth was systematically scanned from the pulpal floor to the apex in axial, sagittal, and coronal planes. Patient sex was also recorded.

In axial sections, a narrow ribbon- or fin-shaped radiolucent connection between the MB and ML canals was identified as an isthmus. To distinguish isthmuses from imaging artifacts, only structures showing continuity toward the apex and running parallel to the pulpal floor were considered true isthmuses. In contrast, artifacts typically appeared in single slices, lacked continuity, and exhibited random orientations or abrupt intensity variations compared with true anatomical features.

An MMC was recorded when a distinct, round or oval radiolucent structure was observed between the MB and ML canals, regardless of the presence of an isthmus.

### Morphometric analysis and canal classification

Observers examined CBCT images in axial, sagittal, and coronal planes to accurately identify canal morphology. All morphometric evaluations were performed using dynamic multiplanar reconstruction. MMC configurations were categorized into four groups based on the classification system proposed by Barros costa et al*.* [13], as modified for CBCT assessment.

**Fig. 1:**
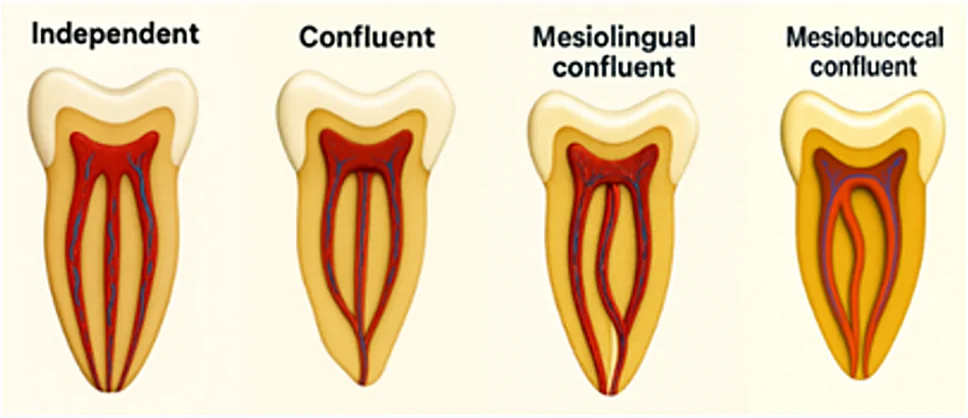
Classification of the morphological configurations of the middle mesial canal (MMC)

- **Independent type:** The MMC originates from a separate canal orifice and terminates at a distinct apical foramen.
- **Confluent type:** The MMC originates from a separate orifice and merges apically with either or both the mesiobuccal (MB) and mesiolingual (ML) canals.
- **ML-confluent type:** The MMC originates from a separate orifice and merges solely with the ML canal.
- **MB-confluent type:** The MMC originates from a separate orifice and merges solely with the MB canal (Fig. 1).

### Morphometric measurements

#### Vertical distance of mesial canals from the pulpal floor

The vertical distances between the pulpal floor and the mesial canals (MB, ML, and MMC) were measured in the sagittal plane as the distance from the first axial slice in which each canal was identifiable to the pulpal floor. The pulpal floor was defined as the lowest boundary of the pulp chamber, surrounded by a highly radiodense dentinal wall marking the transition to the root canal system. To ensure accuracy and standardization, each tooth was aligned along its long axis in the sagittal plane. Using the corresponding axial and sagittal references, the vertical distances between the canal orifices and the pulpal floor were measured consistently across all samples. This approach ensured reproducibility of measurements by applying identical anatomical reference criteria for each case.

### Inter-canal distance measurements

In the axial slice where the mesial canal orifices (MB, ML, and MMC) were first identified, the geometric centers of each orifice were determined. Linear distances were measured from the center of the MMC orifice to those of the MB and ML orifices, respectively. Additionally, the distance between the MB and ML canal orifices was recorded.

These measurements were used to analyze potential associations between the presence of an MMC and the inter-orifice distance of the MB–ML canals, as well as the spatial relationship of the MMC to its neighboring canals.

### Buccolingual and mesiodistal distance measurements

The buccolingual (BL) and mesiodistal (MD) widths of the mesial root were measured in an anatomical reference plane parallel to the pulpal floor—corresponding to the level at which the MB and ML canal orifices were simultaneously visualized in the same axial section. This plane also corresponds to the clinical reference level typically observed during endodontic access cavity preparation. All measurements were performed independently by two experienced endodontists. Each specimen was aligned along its mesial root long axis in the sagittal plane to ensure positional standardization. The morphological flatness index of the mesial root was calculated as the ratio of the buccolingual to the mesiodistal width (BL/MD) [14].

### Statistical analysis

The association between categorical variables was analyzed using the Chi-square test. For data with small expected frequencies (< 5), Fisher’s exact test was applied. The Shapiro–Wilk test and Q–Q plots were used to assess normality, while the F-test and Levene’s test evaluated homogeneity of variances. Because the assumptions of parametric tests were not met, non-parametric alternatives were employed: the Mann–Whitney U test (Wilcoxon rank-sum test) for two-group comparisons and the Kruskal–Wallis test for multiple-group comparisons.

*P*-values < 0.05 were considered statistically significant. Statistical analyses were performed using RStudio (version 2024.04.2) [22] with the following R packages: *summarytools* [23] for descriptive summaries and frequency tables, *stats* [24] for parametric assumption testing and inferential analysis, and *ggstatsplot* [25] for data visualization. Effect size of Mann–Whitney U test is commonly classified as small (≥ 0.11), medium (≥ 0.28), or large (≥ 0.43) based on rank-biserial correlation (|r_rb_|). Effect size of Kruskal–Wallis test is classified as small (≥ 0.01), Medium (≥ 0.06) and Large (≥ 0.14) based on ordinal epsilon-squared (ε_2r_) [26].

## Results

Among the participants, 61.55% were female (*n* = 1846) and 38.45% were male (*n* = 1153). The majority of mandibular first molars (75.5%) did not present a middle mesial canal (MMC), whereas 24.5% (*n* = 735) exhibited its presence. A statistically significant difference was observed between sexes (*p* < 0.001): MMCs were found in 20.7% (*n* = 383) of females and 30.5% (*n* = 352) of males (Table 1).

**Table 1:** Distribution of midmesial canal presence by gender

	Midmesial canal			
	Absent	Present	Total	*P* value
	N (%)	N (%)	N	
Female	1463 (79.3%)	383 (20.7%)	1846 (61.55%)	1.38E-09
Male	801 (69.5%)	352 (30.5%)	1153 (38.45%)	***
Total	2264 (75.5%)	735 (24.5%)	2999 (100%)	

Among the teeth presenting a middle mesial canal (MMC), the **most prevalent configuration** was the **MB-confluent type,** observed in **572 samples (77.8%),** followed by the **ML-confluent configuration** in **83 samples (11.3%).** A smaller proportion exhibited the general confluent type **(72 samples, 9.8%),** while the **independent MMC configuration** was the least frequent, detected in only **8 samples (1.1%).**

When stratified by sex, the proportions of **independent, ML-confluent**, and **confluent** MMCs were lower in females (0.8%, 6.0%, and 6.3%, respectively) than in males (1.4%, 17.0%, and 13.6%, respectively). The **MB-confluent MMC** was the most frequent configuration in both sexes, but occurred at a higher rate in females **(86.9%)** than in males **(67.9%).** Although the proportion of independent MMCs was relatively low in both sexes, it was slightly higher in males than in females.

The differences in root canal configuration distribution between males and females were **statistically significant (p < 0.001)** (Table 2).

**Table 2:** Distribution of root canal configuration by gender for midmesial canals

	Root canal configuration					
	Confluent	Independent	MB confluent	ML confluent	Total	P value
	N (%)	N (%)	N (%)	N (%)	N	
Female	24 (6.3%)	3 (0.8%)	333 (86.9%)	23 (6.0%)	383	5.73E-09
Male	48 (13.6%)	5 (1.4%)	239 (67.9%)	60 (17.0%)	352	***
Total	72 (9.8%)	8 (1.1%)	572 (77.8%)	83 (11.3%)	735	

In the overall analysis, regardless of any grouping variable, the **mean distance between the MB and ML canal orifices** was found to be **3.76 mm** (Table 3). Furthermore, the MB–ML inter-orifice distances (in mm) were compared based on (a) the **presence or absence of an MMC,** and (b) the **different MMC configurations.**

**Table 3:** Distance between the MB and ML canal orifices in mandibular first molars

Variable	N	Min	Max	Median	Mean	SD
MB-ML	2999	3.34	3.88	3.79	3.759	0.096

The **Mann–Whitney U test** revealed a statistically significant difference in MB–ML distance between teeth with and without an MMC **(p < 0.001).** The distance was greater in teeth **without** an MMC (median = 3.81 mm) than in those **with** an MMC (median = 3.62 mm). This difference demonstrated a **large effect size (r**_**rb**_** = 0.98,** 95% CI [0.98, 0.99]**)** and is considered **clinically relevant.**

**Fig. 2:**
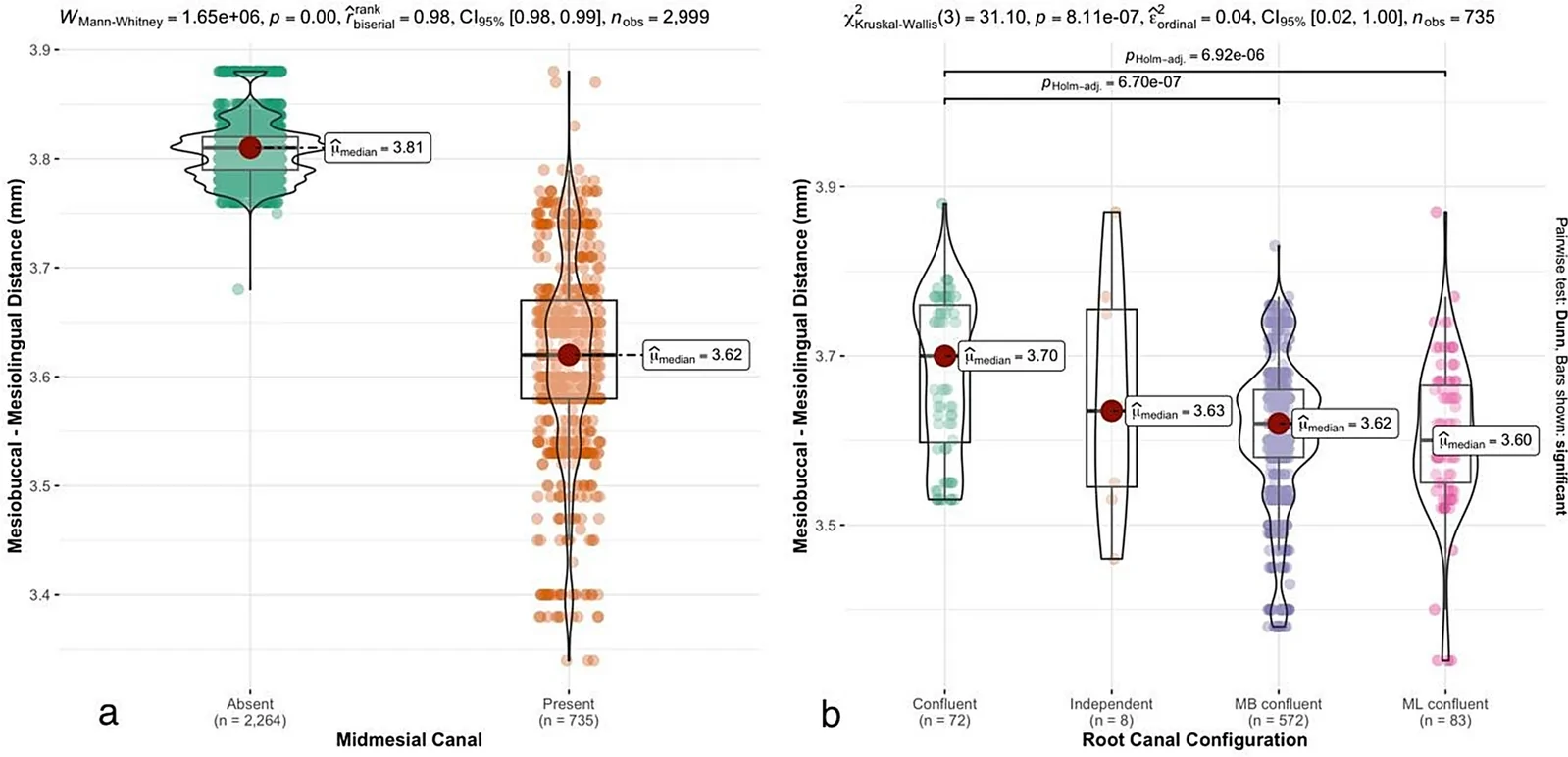
**a** Comparison of MB–ML inter-orifice distances (mm) between samples with and without an MMC. **b** Comparison of MB–ML inter-orifice distances (mm) among samples with different MMC configurations

The **Kruskal–Wallis test** indicated significant differences among the four canal configuration groups (p < 0.001, ε^2^ = 0.04, 95% CI [0.02, 1.00]), and post hoc pairwise comparisons confirmed specific intergroup differences (Fig. 2b). The median MB–ML distances for each configuration were as follows: **confluent type**—3.70 mm, **independent type**—3.63 mm**, MB-confluent type**—3.62 mm, and **ML-confluent type**—3.60 mm.

Both the **MB-confluent** and **ML-confluent** canal types exhibited significantly smaller MB–ML distances than the general confluent type. The presence of the MMC and variations in root canal configuration were associated with statistically significant differences in the MB–ML distance (Fig. 2a).

The distances between the MMC and the adjacent MB or ML canals were compared. The Mann–Whitney U test revealed a statistically significant difference between the MMC–MB and MMC–ML distance groups (*p* < 0.001). The median distance was slightly greater for MMC–ML (median = 1.82 mm) than for MMC–MB (median = 1.80 mm), with a small effect size (*r*_rb_ = − 0.13, 95% CI [− 0.19 − 0.07]).

**Fig. 3:**
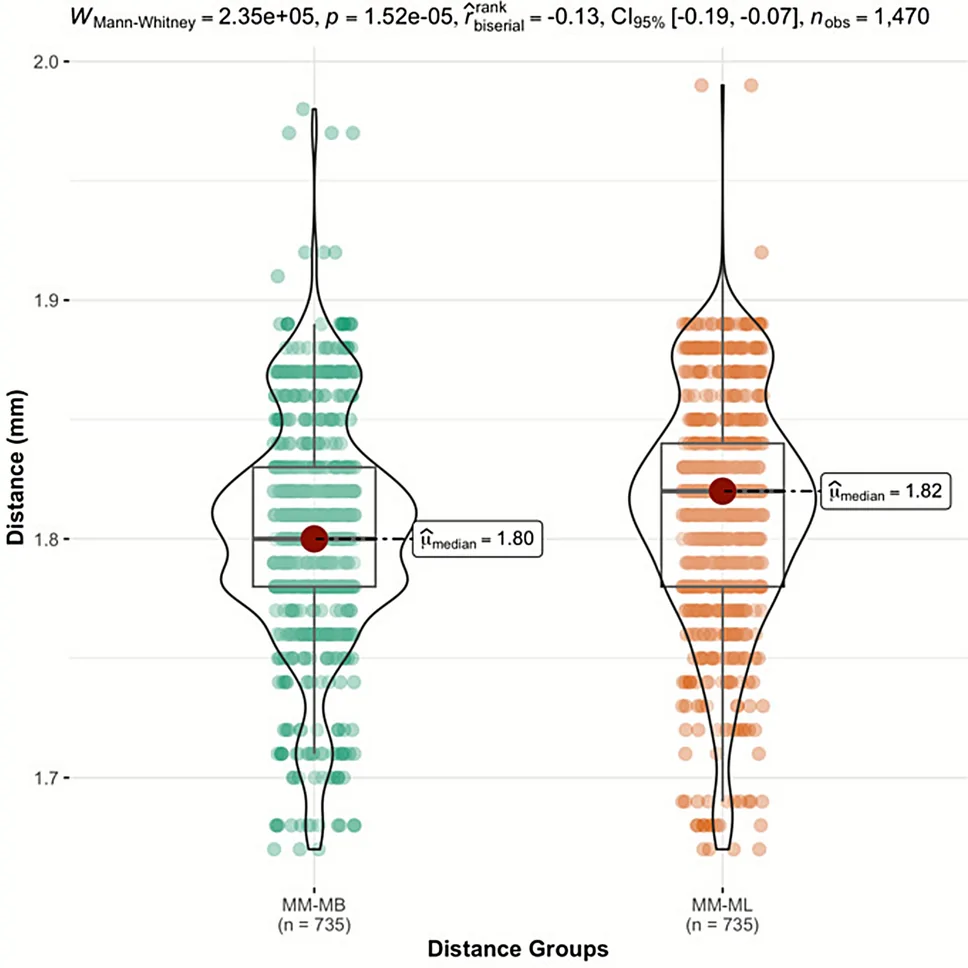
Comparison of MMC–MB and MMC–ML Distances

Although the variation was statistically significant, the magnitude of difference was minimal and not considered clinically meaningful. These findings indicate that the spatial relationship of the MMC with the adjacent MB and ML canals is relatively symmetrical, showing only minor variations in inter-orifice distances (Fig. 3).

When comparing the distances between the MMC and the MB (**a**) and ML (**b**) canals across different root canal configurations, the MMC was found to be closer to the MB canal than to the ML canal. Although this difference was statistically significant according to the Kruskal–Wallis test (*p* < 0.001), the effect size was small (*ε*^2^ = 0.03, 95% CI [0.01, 1.00]).

The median MMC–MB distance showed slight variation among configurations, with values of 1.82 mm (confluent), 1.81 mm (independent), 1.80 mm (MB-confluent), and 1.80 mm (ML-confluent). Pairwise post hoc comparisons revealed a statistically significant difference only between the confluent and MB-confluent groups.

Similarly, the root canal configuration had a significant effect on the MMC–ML distance (*p* < 0.001), with a slightly larger effect size observed (*ε*^2^ = 0.05, 95% CI [0.03, 1.00]). The median MMC–ML distance was greatest in the confluent configuration (1.87 mm), followed by the independent (1.82 mm), MB-confluent (1.82 mm), and ML-confluent (1.80 mm) groups. Pairwise comparisons demonstrated statistically significant differences among several configurations.

**Fig. 4:**
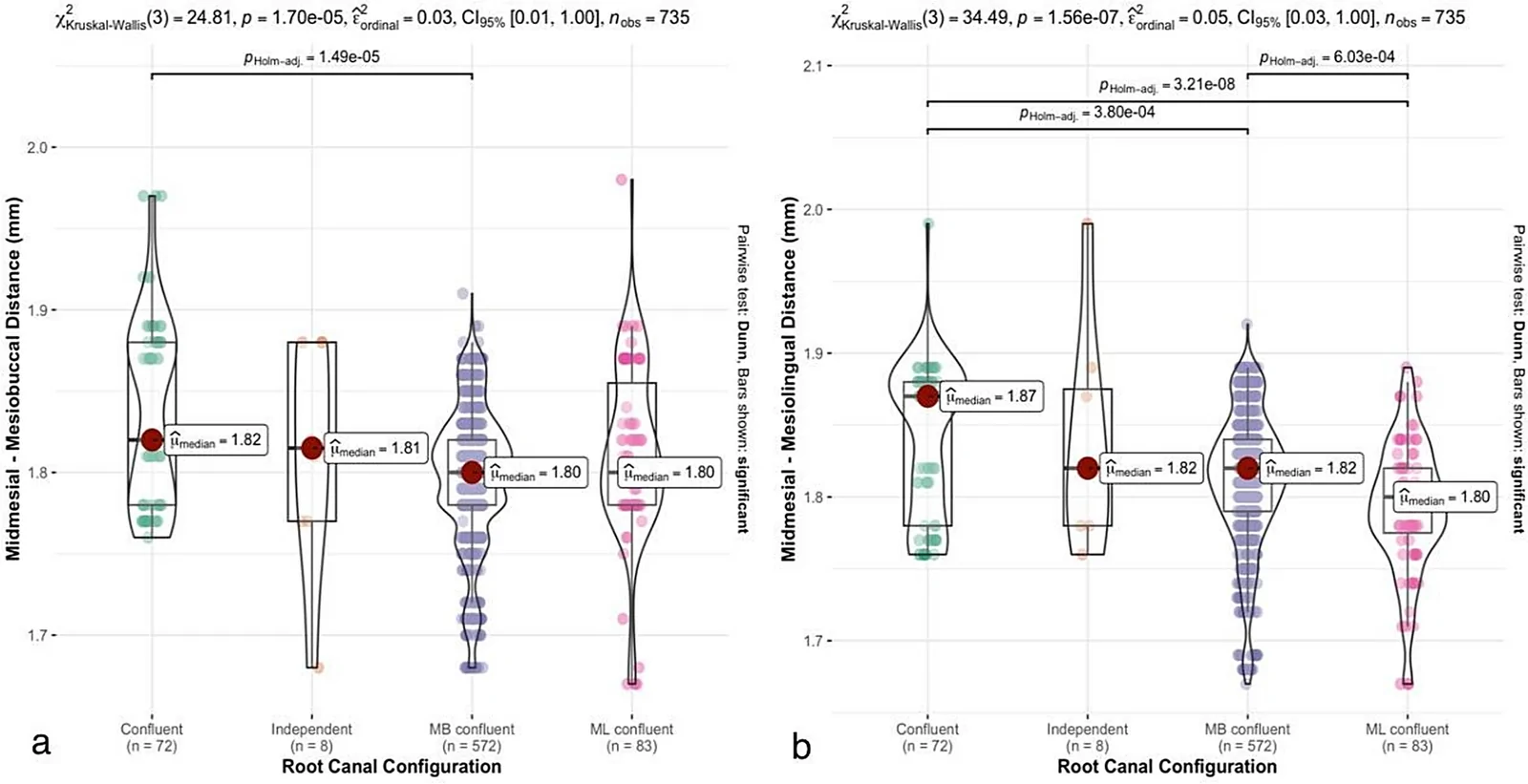
**a** Comparison of MMC–MB distances (mm) across different MMC configurations. **b** Comparison of MMC–ML distances (mm) across different MMC configurations

These results indicate that variations in root canal configuration are associated with measurable differences in MMC–MB and MMC–ML distances, suggesting that subtle spatial changes in MMC morphology may influence endodontic procedures and clinical decision-making (Fig. 4).

**Fig. 5:**
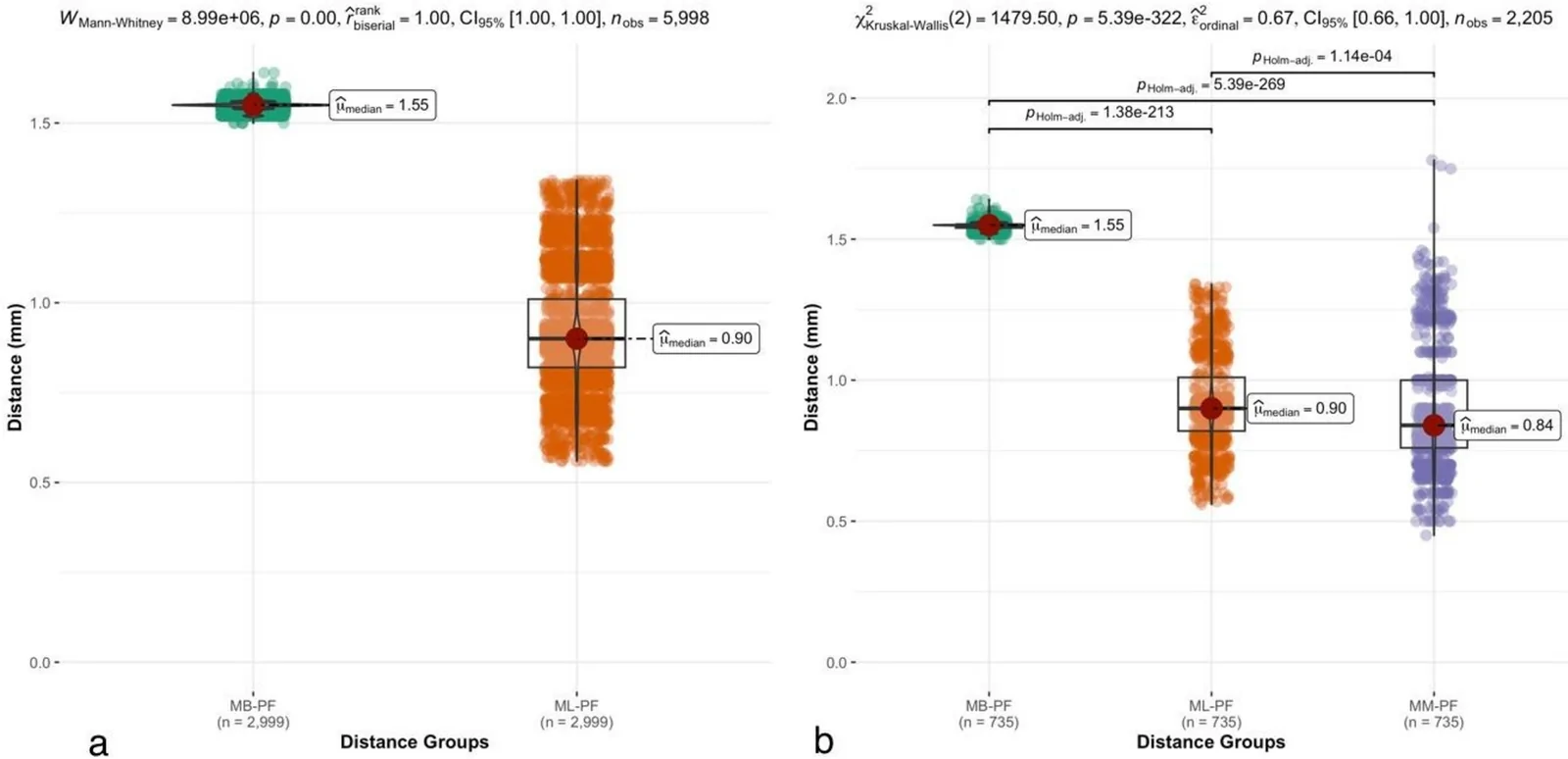
**a** Comparison of MB–PF and ML–PF distances (mm) in the overall sample. **b** Comparison of MB–PF, ML–PF, and MMC–PF distances (mm) in samples with MMCs

The distance between the MB canal orifice and the pulpal floor (MB–PF) was consistently greater than both the ML–PF and MMC–PF distances. In the overall sample, the median MB–PF distance (1.55 mm) was significantly greater than the median ML–PF distance (0.90 mm), as confirmed by the Mann–Whitney test (*p* < 0.001). This difference exhibited a large effect size (*r*_rb_ = 1.00, 95% CI [1.00, 1.00]) (Fig. 5a).

Within the subgroup of teeth exhibiting an MMC, the Kruskal–Wallis test demonstrated a significant difference among groups (*p* < 0.001), revealing a large effect size (*ε*^2^ = 0.67, 95% CI [0.66, 1.00]). Post-hoc analysis further showed that the MB–PF distance (1.55 mm) was significantly greater than both the ML–PF distance (0.90 mm) and the MMC–PF distance (0.84 mm) (Fig. 5b).

However, no statistically significant differences were observed between MB–PF and ML–PF distances when comparing teeth with and without an MMC, nor across different MMC configurations.

No statistically significant differences were found in MB–PF or ML–PF distances between teeth with and without an MMC. In addition, these distances did not vary significantly across different root canal configurations.

**Fig. 6:**
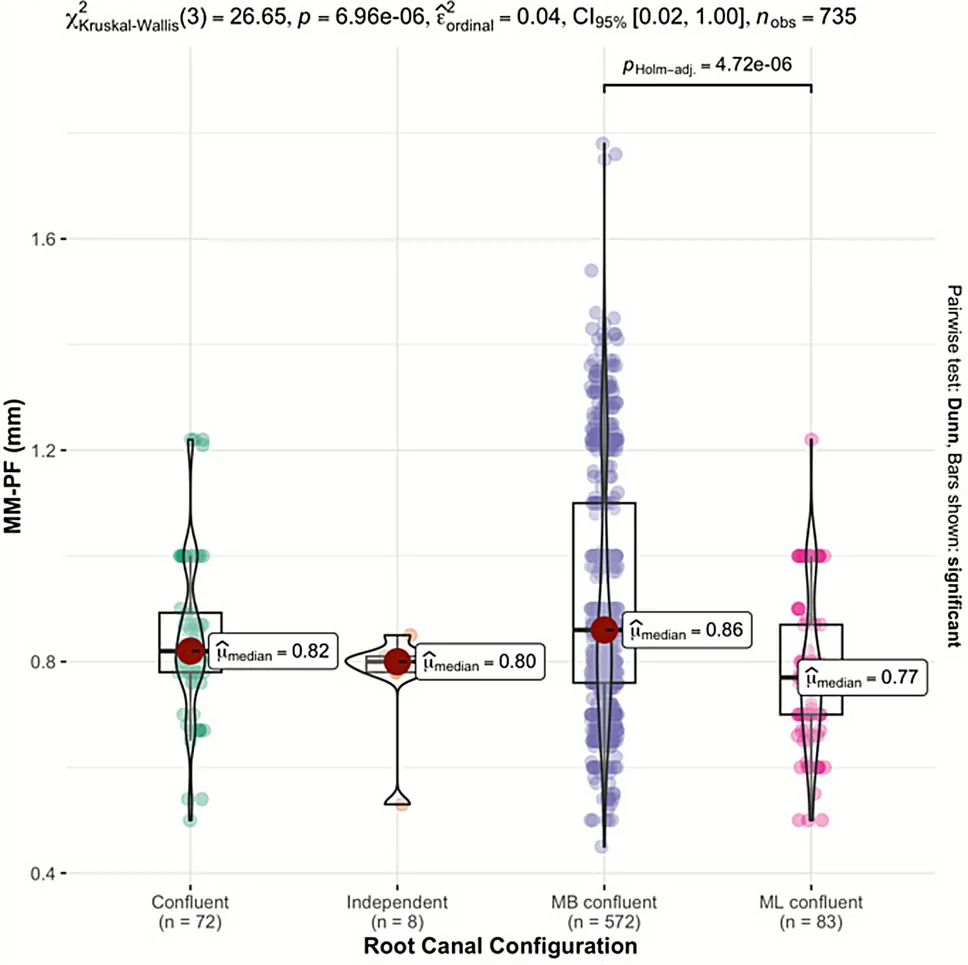
Comparison of the distances between the MMC and the pulpal floor (PF) across different root canal configurations

However, analysis of the distance between the MMC and the pulpal floor (MMC–PF) revealed statistically significant differences among root canal configuration types (*p* < 0.001) (Fig. 6). Although a general difference was observed across configurations, the canal type explained only a small proportion of this variance (*ε*^2^ = 0.04, 95% CI [0.02, 1.00]).

Specifically, MB-confluent canals demonstrated a significantly greater MMC–PF distance (median = 0.86 mm) compared with ML-confluent canals (median = 0.77 mm) (Fig. 6).

Regardless of grouping variables, the mean buccolingual (BL) width of mandibular first molars (MFMs) was 9.33 mm, while the mean mesiodistal (MD) width was 3.09 mm. A significant difference in BL width was observed according to the presence of an MMC; teeth without an MMC exhibited a greater BL width (p < 0.001) (r_rb_ = 0.58, 95% CI [0.55, 0.61]).

**Fig. 7:**
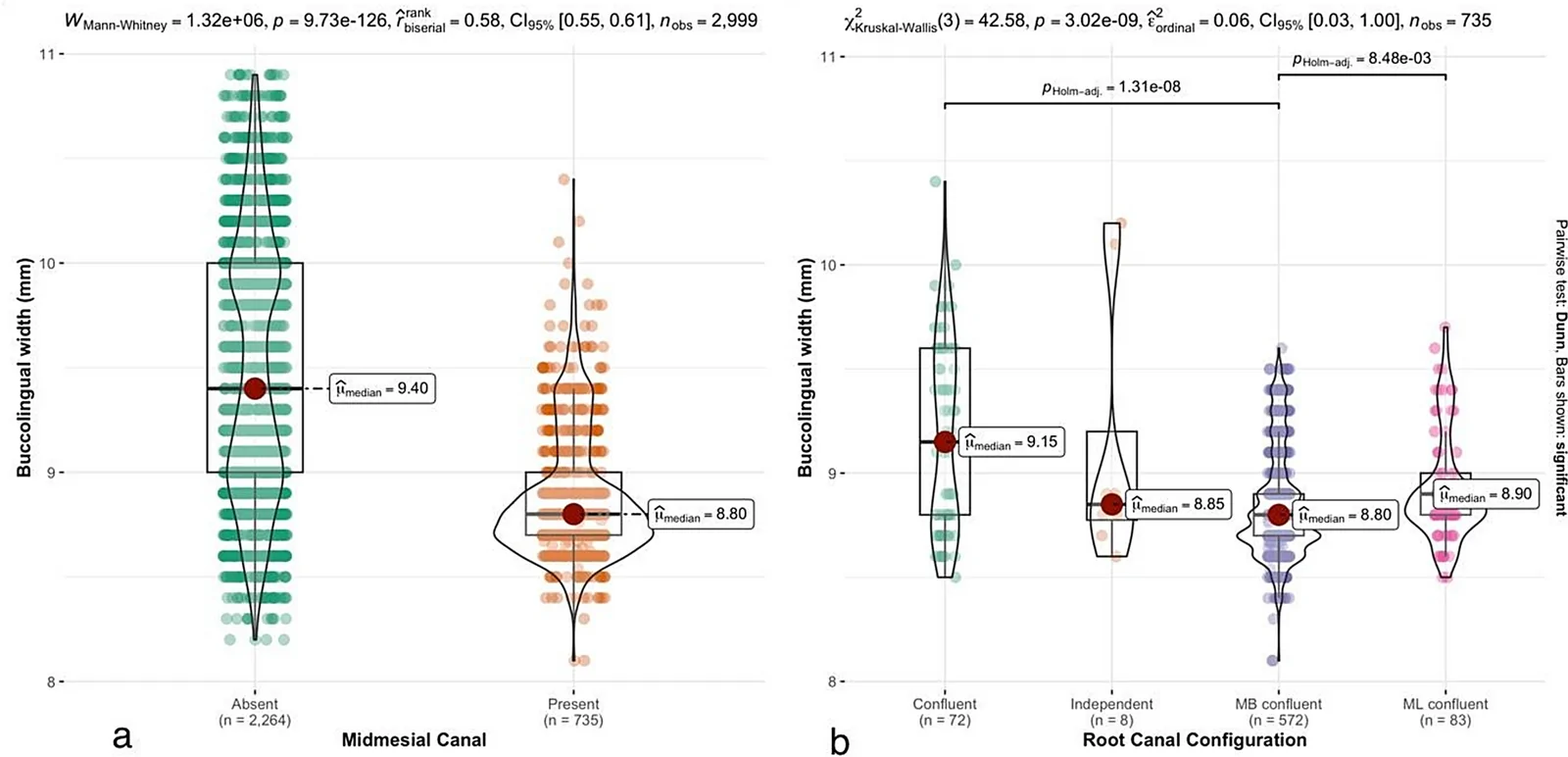
**a** Comparison of buccolingual (BL) width between samples with and without an MMC. **b** Comparison of BL width among samples with different MMC configurations

Among teeth presenting an MMC, the BL width also varied significantly across different root canal configurations (p < 0.001) (ε^2^ = 0.06, 95% CI [0.03, 1.00]). Specifically, teeth with the confluent configuration demonstrated the largest BL width, whereas those with the MB-confluent configuration showed the smallest BL width (Fig. 7).

The mesiodistal (MD) width was slightly affected by the presence of an MMC, resulting in significantly wider teeth (*p* < 0.001) (*r*_rb_ = − 0.10, 95% CI [− 0.15 − 0.05]). Among teeth with an MMC, the MD width also varied significantly across different root canal configurations (*p* < 0.001) (*ε*^2^ = 0.03, 95% CI [0.01, 1.00]).

**Fig. 8:**
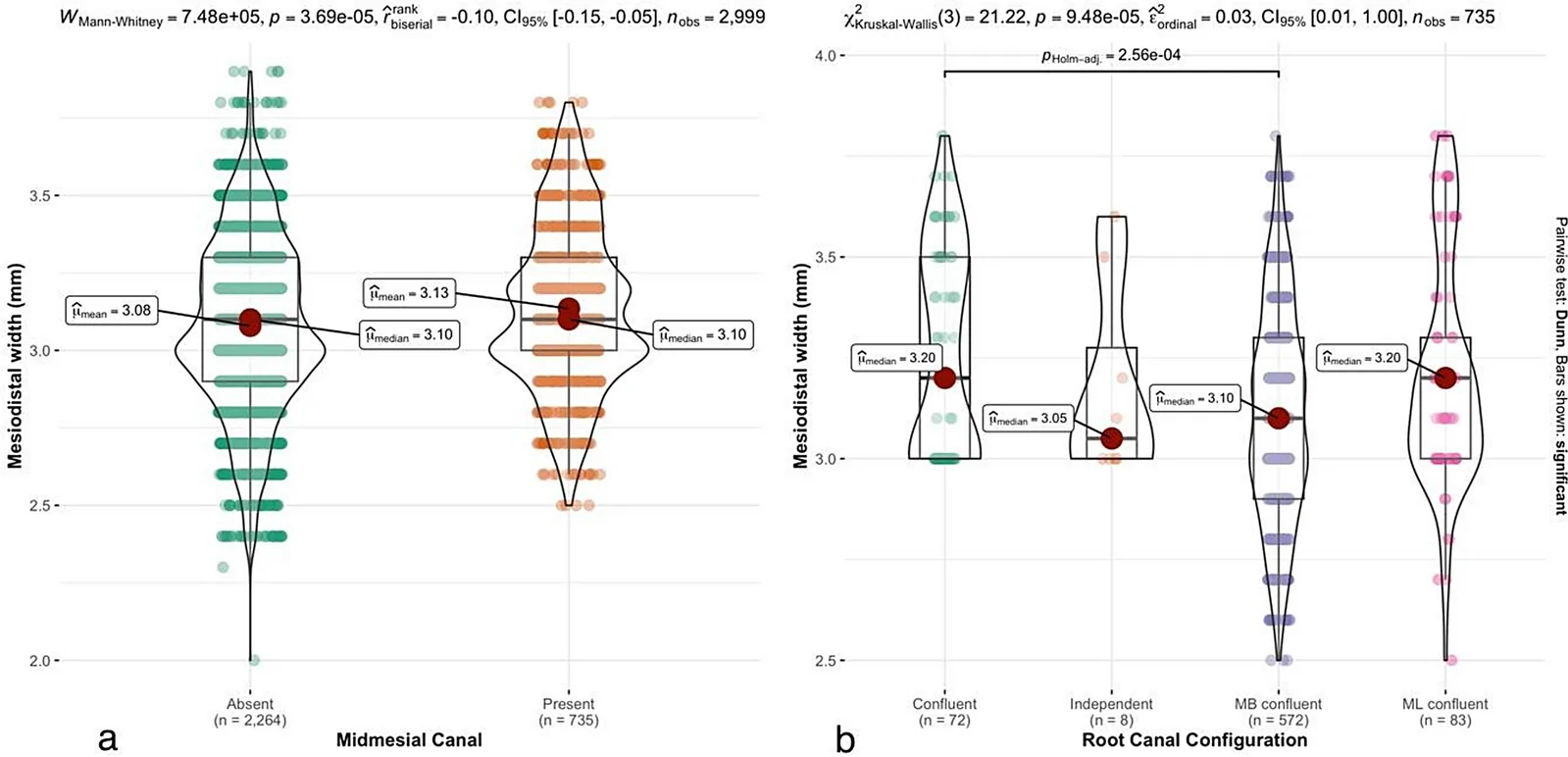
**a** Comparison of mesiodistal (MD) width between samples with and without an MMC. **b** Comparison of MD width among samples with different MMC configurations

Specifically, the confluent configuration exhibited a greater MD width compared with both the MB-confluent and independent types (Fig. 8).

**Fig. 9:**
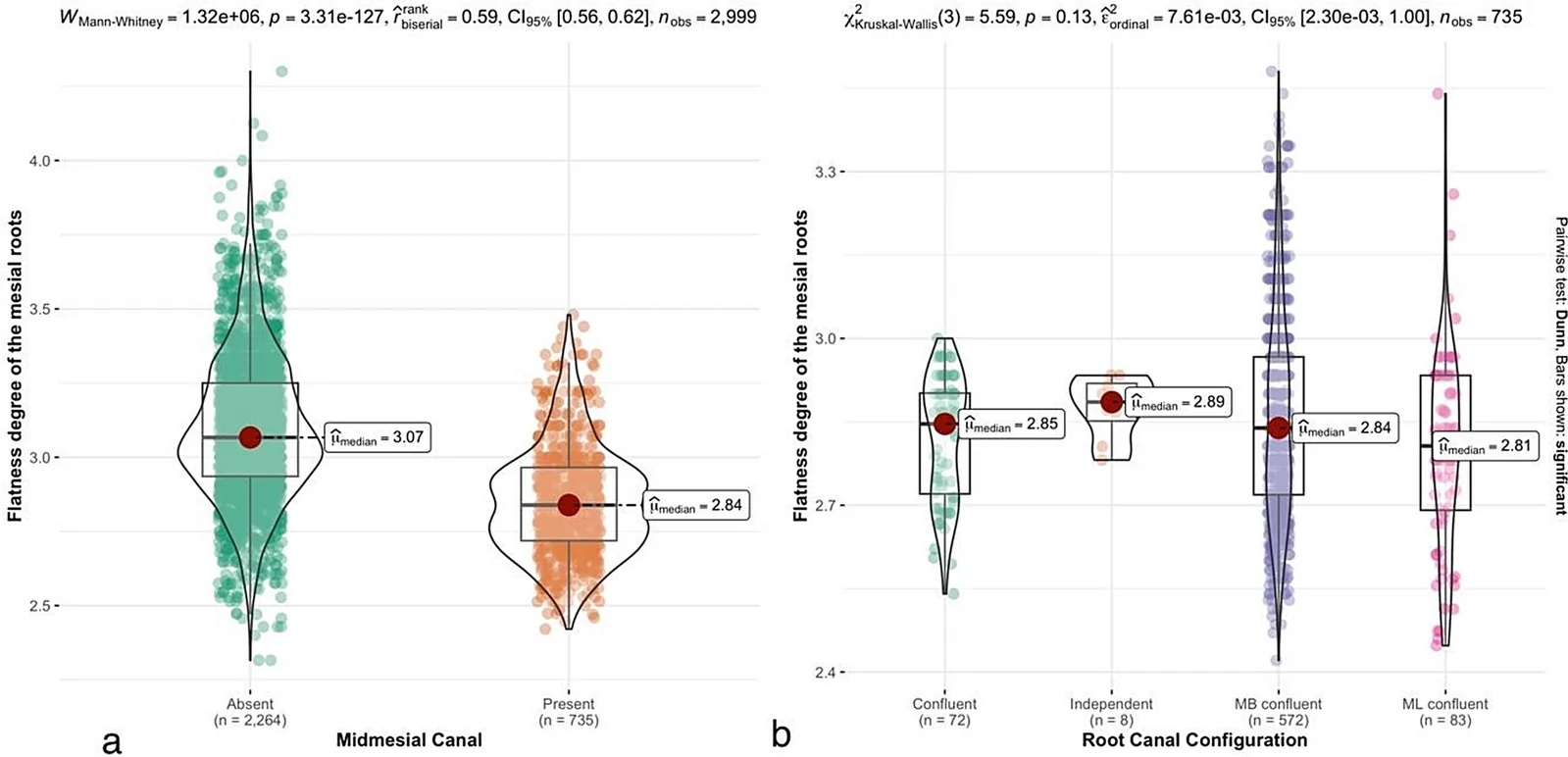
**a** Comparison of the degree of flatness of mesial roots between samples with and without an MMC. **b** Comparison of the degree of flatness of mesial roots among samples with different MMC configurations

When analyzing the relationship between root canal anatomy and the degree of flatness of the mesial root—calculated as the ratio of buccolingual to mesiodistal width (BL/MD) it was evident that roots without an MMC exhibited a significantly higher flatness ratio (median = 3.07) compared with those with an MMC (median = 2.84) (Fig. 9a) (*r*_rb_ = 0.59, 95% CI [0.56, 0.62]).

In contrast, no statistically significant differences were observed among different root canal configuration types (*p* = 0.13), although slight variations in flatness ratio were noted: confluent = 2.85, independent = 2.89, MB-confluent = 2.89, and ML-confluent = 2.81. The very small effect size suggests that the canal configuration exerts no clinically meaningful influence on the overall flatness of the mesial root (Fig. 9B) (*ε*^2^ = 0.008, 95% CI [0.002, 1.00]).

## Discussion

The prevalence of the middle mesial canal (MMC) in mandibular first molars (MFMs) has been reported to vary widely, ranging from 0.26 to 53.8% in previous studies [10]. A study conducted in the Turkish population reported a prevalence of 15.58% [27]. The prevalence observed in the present study (24.5%) is higher than that reported in earlier Turkish studies but still falls within the overall range documented in the literature. This finding indicates that, in the Turkish population, an MMC may be encountered in approximately one out of every four cases, a clinically significant observation given that failure to detect this canal may result in endodontic treatment failure. Differences in prevalence values are commonly attributed to ethnic variations; however, even among individuals of the same ethnicity, variations can arise due to factors such as sample size, number and experience of observers, and differences in imaging modalities and their technical parameters.

Although CBCT is widely used in clinical practice, it is not considered the gold standard imaging modality. Micro-CT and nano-CT techniques provide higher spatial resolution and allow for more precise evaluation of MMC morphology; however, due to their high radiation dose and prolonged scanning time, their use is limited to in vitro conditions [13]. In contrast, CBCT offers greater clinical accessibility and chairside applicability, which justified its use in the present study. Previous investigations have shown that voxel sizes of 200 μm or smaller are adequate for analyzing dental anatomy [18, 28, 29]. CBCT systems are classified based on FOV dimensions into large (> 100 cm^2^), medium (40–100 cm^2^) and limited (< 40 cm^2^) volumes. While radiation dose increases directly with FOV size, image resolution decreases inversely due to the effect of scatter radiation. Since endodontics typically targets localized regions of interest (ROI), limited FOV protocols offering reduced artifact risk, superior spatial resolution, and lower radiation exposure are the primary preference [30]. However, larger FOV scans may be indicated in cases where lesion borders extend beyond the localized area or systemic etiology is suspected (17). Accordingly, the imaging parameters in the current study were determined in adherence to the ALADA (As Low As Diagnostically Acceptable) principle. The selected 8 × 8 cm FOV and 200 μm voxel size provided sufficient diagnostic resolution for endodontic evaluation without creating an unnecessary radiation burden [31].

Regarding gender distribution, some studies have reported a higher MMC prevalence in males, while others have found higher rates in females [26, 32]. In this study, MMCs were more frequently observed in males; however, conflicting findings and meta-analyses suggest no consistent relationship between MMC occurrence and gender [33].

Although the classification system proposed by Pomeranz et al. was initially considered, this study adopted the Barros-Costa et al. CBCT-based classification [13] due to its improved ability to distinguish true canals from isthmus formations, which may significantly influence reported prevalence. Literature review revealed the mesiobuccal (MB)-confluent configuration as the most frequent and the independent configuration as the least common type [34, 35]. In the present study, the MB-confluent type demonstrated the highest prevalence (78.8%), while the independent type was the least frequent (1.1%), consistent with existing reports. The predominance of the MB-confluent configuration supports the hypothesis that the MMC tends to course buccally, a morphological trait that may aid clinicians in precurving files and planning access cavity direction.

Studies investigating morphometric relationships between MMC presence and position and buccolingual (BL) or mesiodistal (MD) measurements are limited. One study reported that teeth with MMCs had greater BL width but similar MD width compared with those without MMCs, resulting in no significant correlation between MMC presence and MD width [13]. Another study found no significant relationship between MMC presence and either morphometric parameter [14]. In contrast, the present study observed a shorter BL width and a greater MD dimension in teeth with MMCs. These discrepancies highlight the need for future research with larger sample sizes.

The BL/MD ratio serves as a critical parameter for identifying round versus oval canal morphology and assessing root canal shaping efficiency. Previous studies have demonstrated that oval and long-oval canals are prone to incomplete instrumentation, leaving uncleaned dentin surfaces. In the present study, the lower BL/MD ratio in MMC-containing teeth indicates a flatter root morphology. However, since both groups exhibited ratios within the 2–3 range, the canals can be classified as long oval, suggesting limited contact between instruments and canal walls, emphasizing the importance of irrigant activation in achieving optimal cleaning efficacy [36, 37].

Similarly, the relationship between MB–ML interorifice distance and MMC presence was evaluated. The correlation between these parameters remains controversial in the literature. Studies by Akbarzadeh et al. and Weinberg et al. reported a negative correlation between MMC occurrence and MB–ML distance, consistent with our findings [38, 39]. Conversely, some researchers have found a positive correlation, suggesting an increased likelihood of MMC presence as the MB–ML distance increases [13, 40]. Such inconsistencies may arise from ethnic variations, methodological differences, and inconsistent evaluation criteria. One morphometric parameter that may facilitate MMC detection is the distance of the MMC orifice from the MB and ML canal orifices. In the present study, the distance between MMC and MB orifices was shorter, aligning with the predominance of the MB-confluent configuration in our sample. However, due to the small effect size, this difference is unlikely to be of clinical significance. Previous studies have reported that the MMC orifice is typically located near the center of the mesial root or closer to the mesiolingual canal [35, 41].

With respect to vertical positioning, clinical and micro-CT studies have shown that MMCs are generally detectable within 2 mm of troughing below the cementoenamel junction (CEJ). In a micro-CT study by Keleş et al., 77.41% of MMC orifices were located at the CEJ level, indicating that no troughing was necessary in those cases. The same study reported that 5.38% of orifices were 1 mm, 9.69% were 2 mm, and 7.52% were ≥ 2 mm below the CEJ [40, 41]. In contrast, CBCT-based studies have found MMC orifices approximately 3–4 mm apical to the pulpal floor [18, 39]. In the present study, the larger MB–PF distance supports the occlusal proximity of the MB pulp horns, while the greater ML–PF compared with MMC–PF distance suggests that performing a slight troughing after locating the MB and ML canals is a clinically effective approach for MMC detection. These findings indicate that predicting MMC presence cannot rely on a single morphometric indicator but instead requires multi-parametric assessment.

When interpreting the results of the present study, several limitations should be considered. First, due to the retrospective nature of the analyzed CBCT data, the scans were not originally acquired for the specific purpose of identifying the middle mesial canal (MMC). The voxel size of 200 μm used in this study may have partially limited the visualization of fine anatomical structures. Previous studies have demonstrated that smaller voxel sizes (< 200 μm) increase diagnostic sensitivity, particularly for identifying fine anatomical details such as MMCs or root canals of mandibular incisors [42–44]. In addition, although a contemporary CBCT-based classification system was used to evaluate MMC morphology, this system does not distinguish whether confluent canal types redivide into two separate canals in the apical region or terminate in separate apical foramina. This limitation may restrict a detailed understanding of the apical configurations of MMCs. Another methodological limitation is that bilateral occurrence of MMCs was not analyzed in patients with both right and left mandibular first molars. Consequently, teeth were evaluated as independent units, and potential intra-patient correlation was not taken into account, which may have influenced the reported prevalence values.

Furthermore, as this study was conducted exclusively in a Turkish population, the generalizability of the findings to other ethnic groups may be limited. The lack of age matching between study groups also represents a limitation. The effect of age on MMC prevalence remains controversial in the literature, with conflicting views suggesting either reduced canal visibility due to calcification or, conversely, increased canal numbers due to isthmus differentiation [45, 46]. However, most recent CBCT-based studies have not demonstrated a statistically significant association between age and the presence of MMCs. Therefore, although the potential influence of age cannot be completely excluded, further studies with age-matched groups and larger sample sizes are required to clarify this issue.

## Conclusion

This CBCT-based study comprehensively evaluated the prevalence, morphological configurations, and morphometric relationships of the middle mesial canal (MMC) in mandibular first molars within a Turkish population. The observed prevalence of 24.5%, predominantly represented by the mesiobuccal-confluent configuration, underscores the need for careful clinical assessment during endodontic treatment.

The findings indicate that morphometric parameters such as a shorter buccolingual distance and a greater mesiodistal width are associated with the presence of an MMC, which may serve as diagnostic indicators for clinicians. Furthermore, the deeper pulpal location of the MMC orifice relative to the MB and ML canals suggests that troughing after locating the main mesial canals is an effective clinical approach for MMC detection.

In conclusion, this study contributes to a better understanding of the anatomical and clinical characteristics of the MMC and provides valuable insights to enhance the accuracy and success of endodontic procedures in mandibular first molars.

## Data Availability

The data supporting the findings of this study are available from the corresponding author upon reasonable request.
